# Human Mitochondrial DNA: Particularities and Diseases

**DOI:** 10.3390/biomedicines9101364

**Published:** 2021-10-01

**Authors:** Mouna Habbane, Julio Montoya, Taha Rhouda, Yousra Sbaoui, Driss Radallah, Sonia Emperador

**Affiliations:** 1 Laboratoire Biologie et Santé, Faculté des sciences Ben M’Sick, Hassan II University of Casablanca, Sidi Othman, Casablanca 20670, Morocco; taha613@hotmail.com (T.R.); driss.radallah@gmail.com (D.R.); 2 Departamento de Bioquímica, Biología Molecular y Celular, Universidad de Zaragoza, C/Miguel Servet, 177, 50013 Zaragoza, Spain; jmontoya@unizar.es (J.M.); seortiz@unizar.es (S.E.); 3 Instituto de Investigación Sanitaria (IIS) de Aragón, Av. San Juan Bosco, 13, 50009 Zaragoza, Spain; 4 Centro de Investigaciones Biomédicas en Red de Enfermedades Raras (CIBERER), Av. Monforte de Lemos, 3-5, 28029 Madrid, Spain; 5 Département de Biologie, Faculté des Sciences Ain Chock, Hassan II University of Casablanca, Casablanca 20000, Morocco; yousra.sbaoui@gmail.com

**Keywords:** mitochondrial diseases, mtDNA, mutation, molecular diagnosis

## Abstract

Mitochondria are the cell’s power site, transforming energy into a form that the cell can employ for necessary metabolic reactions. These organelles present their own DNA. Although it codes for a small number of genes, mutations in mtDNA are common. Molecular genetics diagnosis allows the analysis of DNA in several areas such as infectiology, oncology, human genetics and personalized medicine. Knowing that the mitochondrial DNA is subject to several mutations which have a direct impact on the metabolism of the mitochondrion leading to many diseases, it is therefore necessary to detect these mutations in the patients involved. To date numerous mitochondrial mutations have been described in humans, permitting confirmation of clinical diagnosis, in addition to a better management of the patients. Therefore, different techniques are employed to study the presence or absence of mitochondrial mutations. However, new mutations are discovered, and to determine if they are the cause of disease, different functional mitochondrial studies are undertaken using transmitochondrial cybrid cells that are constructed by fusion of platelets of the patient that presents the mutation, with rho osteosarcoma cell line. Moreover, the contribution of next generation sequencing allows sequencing of the entire human genome within a single day and should be considered in the diagnosis of mitochondrial mutations.

## 1. Introduction

Mitochondria are intracellular organelles of eukaryotic cells. In humans there can be over 1000 mitochondria per cell and all the mitochondria of one cell are called chondroma. The first mitochondria were observed in muscle in 1857 by Rudolph Albert Von Kölliker, who published numerous books between 1850 and 1890 describing the different cellular structures present in human tissues. It was in the 1960s that Lynn Margulis, an American microbiologist, hypothesized that mitochondria are prokaryotic cells that result from the incomplete endocytosis incorporation of an alpha-proteobacterium by a primary anaerobic protoeukaryotic cell. This allowed the host cell to use oxygen to produce energy and thus survive in aerobic conditions. Today, this theory is widely accepted [1]. Mitochondria therefore have their own DNA (mtDNA), circular and double-stranded, closer to a prokaryotic genome than nuclear DNA, with a genetic code slightly different from the universal genetic code found in the nucleus of eukaryotic cells. They are surrounded by two membranes of different composition: the inner membrane is close to a bacterial membrane in appearance (presence of cardiolipin) and the outer membrane resembles the membrane of a eukaryotic cell. Mitochondria also contain ribosomes called mitoribosomes that are similar to those of bacteria because they are small and vulnerable to antibiotics [2,3,4].

As mentioned above, mitochondria were originally thought to be a proteobacteria that has integrated into an eukaryotic cell by endocytosis. The endosymbiotic origin of mitochondria explains the fact that these organelles have their own genome with a genetic code different from nuclear DNA. However, during evolution, this DNA would have lost most of its genes and these would have been inserted into the nuclear DNA. Its size and gene content vary among different species. In humans, mitochondrial DNA represents about 1% of total cellular DNA (about 1000 to 10,000 copies per cell). The number of copies per mitochondria varies from 5 to 10 [5]. Mitochondria are defined as the power plant of the cell because they provide, by the oxidative phosphorylation system (OXPHOS), almost all the energy that is necessary for the different functions of the cell, this in the form of ATP through a coupling between the respiratory chain and the ATP synthase. These reactions are carried out by enzyme complexes composed of subunits that are encoded by nuclear and mitochondrial DNA [1]. Therefore, more than 200 mutations in mtDNA have been reported [6]. Most mtDNA disorders are heteroplasmic with higher heteroplasmy involved in numerous diseases. However, there are mtDNA disorders that are 100% homoplasmic for pathological mutations Some 100% homoplasmic mtDNA pathological mutations have severe outcomes such as death at young ages [7].

## 2. Particularities: In Eukaryotic Cells, Mitochondria Have Various Characteristics

### 2.1. The Mitochondrial Genome

**Figure 1:**
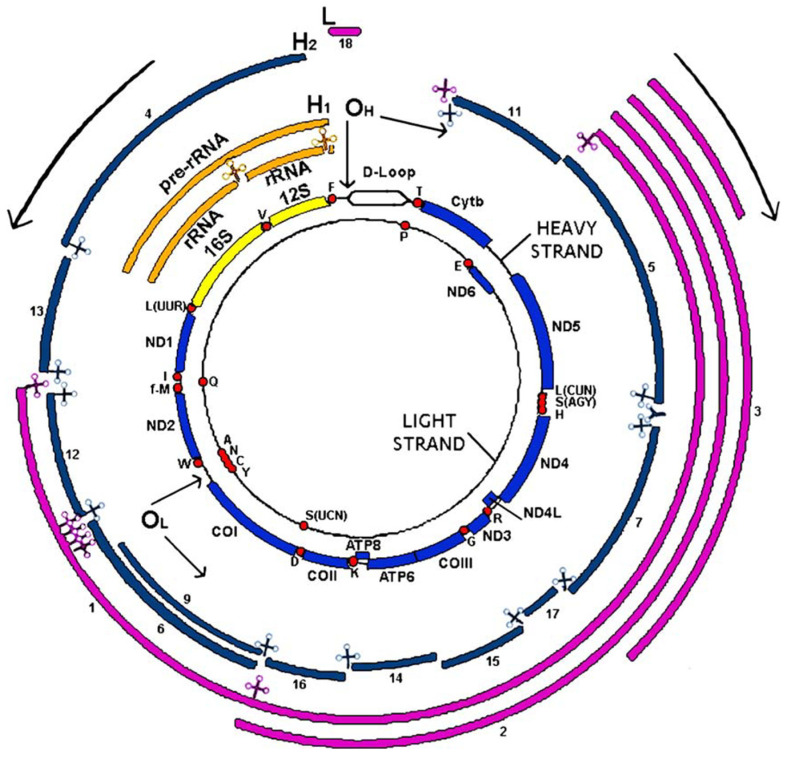
Human mitochondrial DNA: genetics and transcription.

The mitochondrial DNA is a circular molecule of about 16.6 kb (16,569 bp) and unlike the nuclear genome has no introns. The mtDNA is double-stranded. The two strands can be physically separated into a heavy strand (H/heavy) rich in purine bases (G and A) and a light strand (L/light) rich in pyrimidine bases (C and T). Most of the information is found in the heavy strand (H), which encodes 2 *rRNAs* (*12S rRNA* and *16S rRNA*), 14 *tRNAs* and 12 polypeptides, all of which are subunits of the respiratory chain complexes, as follows: six complex I subunits (*ND1*, *ND2*, *ND3*, *ND4*, *ND4*, *ND4L*, *ND5*), one complex III subunit (Cytochrome b), three complex IV subunits (*COI*, *COII*, *COIII*), and two complex V subunits (*ATPase 6* and *ATPase 8*). Complex II, with four subunits, is encoded by the nucleus (Figure 1).

The light strand (L) codes for eight tRNAs and one polypeptide (*ND6,* subunit of complex I). Mitochondrial DNA genes do not have introns and intergenic sequences are absent, except for a 1118 bp regulatory region, the only non-coding region of mtDNA corresponding to the D-Loop displacement loop. This region is located between the genes of *MT-tRNAPhe* and *MT-tRNAPro* and contains the origin of H-strand replication and the promoters of transcription of heavy and light strands [8]. DNA repair systems are essential to maintain the integrity of genetic information since, during the life of an individual, DNA damage may occur. Thus, alterations in DNA sequence and structure can be induced by exogenous chemical or physical factors such as environmental stresses, ionizing or solar radiation (ultraviolet or UV), chemical substances, etc. DNA damage can also be caused by endogenous factors such as intermediates produced during the various metabolic processes. Moreover, DNA undergoes hydrolysis, oxidation and methylation reactions, and errors during replication cycles. These deleterious processes induce modifications (mutations, deletions, rearrangements or modifications of the structure, breaks) that can modify the expression of genes. To correct this damage, there are different DNA repair systems in mammals, specific to the lesions to be corrected.

In mitochondria, the maintenance of mtDNA is essential for the proper functioning of the organelle and the respiratory chain (RC). This requires a fine regulation of the processes that allow its replication, transmission and the maintenance of its integrity and stability. Unlike nuclear DNA, mtDNA is not protected by “histone” proteins and is therefore more susceptible to intrinsic or extrinsic aggression. Its location in the IM near the mitochondrial respiratory chain, which produces free electrons and reactive oxygen species (ROS) by oxidative phosphorylation, is another mutagenic factor. Prolonged exposure to these free radicals leads to an increase in the rate of mutations. There are mitochondrial agents that neutralize ROS produced by the respiratory chain such as catalases or glutathione, although when these antioxidant mechanisms are insufficient, damage to the mtDNA must be corrected. The main consequences of ROS on mitochondrial DNA may be the appearance of oxidized bases, abasic sites or oxidized abasic sites that will cause molecular breaks. Initially, it was assumed that there was no repair mechanism in the mitochondria. Over the past 40 years, several repair mechanisms have been successively identified within mitochondria, which are mediated by enzymes such as those acting in the nucleus. These enzymes are all encoded by the nuclear genome. Among the systems identified in mammalian mitochondria are the BER (Base Excision Repair), SSBR (Single-Strand Break Repair) and DSB (Double-Strand break Repair) systems belonging to the DNA break repair and the MMR (MisMatch Repair). The nucleotide excision repair system has not been identified in mitochondria [9].

The two internal circles represent both mtDNA strands with the encoded genes in yellow (*rRNAs*), red dots (*tRNAs*) and blue (protein coding genes). External circles represent the RNAs transcribed from the heavy strand (in orange or in blue for the *RNAs* derived from the H1 or H2 transcription units) and light strand (in pink). *ND1* to *ND6* are subunits 1–6 of NADH dehydrogenase (complex I); *cyt b*, cytochrome b, is a subunit of complex III; *CO I, CO II* and *CO III* are subunits of cytochrome c oxidase (complex IV) and *ATP6* and *ATP8*, subunits of ATP synthase (complex V). *tRNA* genes are indicated by the one letter code of the corresponding amino acid. OH and OL represent replication origins for the H- and L-strand, respectively, according to the classical model of replication. H1, H2 and L indicate initiation points for the three transcription units of the H- and L-strand, respectively. Arrows at the OH and OL, and in the outside part of the figure, indicate the direction of replication and transcription of both strands [10].

### 2.2. Maternal Origin

Mitochondrial DNA is only of maternal origin. The mother passes her mtDNA to all her children, although only the daughters will pass it on to all members of the next generation. This is due to the high number of mtDNA copies inside the oocyte and the fact that mitochondria in the intermediate region of the sperm are eliminated in the first cell divisions. A few minutes after fertilization, the oocyte initiates an autophagic process: the elements of the sperm are sequestered in vesicles and then eliminated by enzymatic degradation. If the paternal mitochondria are not eliminated and kept in the oocyte, they cause the appearance of so-called mitochondrial diseases. This maternal transmission explains the genetics of diseases related to mitochondrial genome abnormalities [11].

### 2.3. Polyplasmy Heteroplasmy and Homoplasmy

The number of mtDNA copies varies in different tissues: from a very low number of molecules in platelets to more than 100,000 copies in the oocyte. The number of molecules in most tissues varies between 1000 and 10,000 per cell [12]. Within a cell, it is possible that several populations of mtDNA coexist. Indeed, some mtDNA molecules carrying a mutation can coexist with wild type mtDNA molecules. In this case it is called heteroplasmy, while the presence of a unique type of mtDNA is homoplasmy. The proportion of mutated mtDNA in relation to total mtDNA determines the heteroplasmy rate [13].

Thus, when the oocyte develops, blastocyte segmentation and mitotic segregation can lead different heteroplasmy rates to a modification of the mitochondrial genotype between mother cells and daughter cells. This can also lead to a variation in mitochondrial genotype between daughter cells and therefore later to a variation among different tissues [14]. However, mtDNA mutations are generally heteroplasmic because there is coexistence of normal and mutated molecules in the same cell or tissue, so the most affected tissues generally have a high mutation rate [14].

### 2.4. Segregation

During embryogenesis, the nucleus replicates several times and the egg cytoplasm is segmented to form the blastocyst. During this cleavage of the cytoplasmic territory, the distribution of mitochondria within the different blastocyte cells is random. Subsequently, these mitochondria proliferate inside the cytoplasm and each cell divides mitotically, giving rise to two daughter cells [15].

During cell division, the mitochondria of a cell are not evenly distributed in the daughter cells. Thus, from a cell with two types of mitochondrial populations, daughter cells with variable rates of each of the two populations can be obtained. This phenomenon called mitotic segregation explains that an individual can have very variable normal to mutated DNA ratios in different tissues and organs from an egg containing a given proportion of normal and mutated mitochondrial DNA. Regarding the threshold effect, this phenomenon explains the heterogeneity of the clinical expression of diseases related to mitochondrial DNA [16].

### 2.5. High Mutation Rate

The mitochondrial genome is fragile and has an average mutation rate 10–20 times higher than that of the nuclear genome. There are several explanations for this phenomenon: on the one hand, a less effective DNA repair system than in the nucleus and the absence of protection by histone proteins and, on the other, the proximity of the respiratory chain, which generates significant quantities of reactive oxygen species (ROS), exposing the mitochondrial genome to oxidative damage. This is even more important during replication since mitochondrial DNA remains exposed for a long time in single-stranded form, which increases its sensitivity to radical attacks [17]. According to several studies, mitochondrial mutations have also been shown to accumulate with age [11].

### 2.6. Different Types of Mutation

mtDNA can be affected by abnormalities that cause about 20% of mitochondrial diseases. Although it codes for a small number of genes, mutations in this DNA are common. These mtDNA abnormalities are mostly heteroplasmic and maternally transmitted but may in some cases be de novo mutations [18]. The main alterations of mtDNA responsible for mitochondrial diseases are point mutations, deletions, insertion and depletion, related to mutations in nuclear genes involved in the maintenance of mtDNA (Table 1).

Point mutations in mtDNA are, in some cases, associated with characteristic syndromes whose effects depend on the level of heteroplasmy in each tissue. Point mutations can be divided into two groups: mutations in structural genes (coding for *tRNA* and more rarely for mitochondrial *rRNA*) and mutations in the genes encoding the 13 subunits of the respiratory chain.

Unique deletions of mtDNA result in the loss of part of the mtDNA. The size of the deleted fragment varies from 1 to 10 kb and is often between the two origins of mtDNA replication (Origin H and L), involving genes encoding subunits of the respiratory chain and mitochondrial *tRNA*. The deletion boundaries correspond in 85% of the cases to repeated sequences. Unlike multiple deletions, single deletions are sporadic and accidentally appear in the germ line due to an error in mtDNA repair or replication. They are always heteroplasmic. Mothers with this condition rarely transmit this type of abnormality (1 in 24 cases) and recurrence in siblings is low [19]. Further studies show that the size of the deletion and the percentage of heteroplasmy influence the severity and progression of symptoms in these diseases [20]. Mitochondrial pathologies caused by multiple deletions of mtDNA have autosomal dominant Mendelian transmission and appear in an adult subject [20].

Depletion (decrease in the number of copies of mtDNA) occur in the neonatal period or in childhood [21]. They correspond to severe attacks transmitted by autosomal recessive mode. The hepato-cerebral forms, combining early hepatic damage (cytolysis, hepatocellular failure, cholestasis) and axial hypotonia, are due to mutations in the *DGUOK* genes encoding deoxyguanosine kinase, *POLG* encoding gamma mitochondrial DNA polymerase or *MPV17* of unknown function. Mutations are also found in the *TK2* gene encoding thymidine kinase 2, which leads to early and severe myopathy with depletion in skeletal muscle [22].

**Table 1:** The most frequent mitochondrial mutations and associated pathologies.

Pathologies	Mutations	Gene	References
LHON	m.3460G > A	*ND1*	[22]
	m.11778 G > A	*ND4*	[22]
	m.14484T > C	*ND6*	[22]
	m.14459G > A	*ND6*	[22]
NARP	m.8993T > G/C	*ATP6*	[22]
Leigh	m.8993T > G/C	*ATP6*	[22]
	m.9176T > G/C	*ATP6*	[22]
	m.14487T > C	*ND6*	[22]
	ins5537T	*tRNA^Trp^*	[23]
Leigh/LHON	m.13513G > A	*ND5*	[22]
MELAS	m.3243A > G	*tRNA^Leu^* ^(*UUR*)^	[22]
MELAS/DM	m.3271T > C	*tRNA^Leu^* ^(*UUR*)^	[22]
	Hot spot	*tRNA^Leu^* ^(*UUR*)^	[22]
MERRF	m.8344AG	*tRNA^Lys^*	[22]
	Hot spot	*tRNA^Lys^*	[22]
Aminoglycoside-induces non-syndromic deafness	m.1555A > G	*12SrRNA*	[22]
DEAF	m.1546A > T	*12SrRNA*	-
Leigh Disease/Ataxia syndromes/NARP-like disease	m.9185T > C	*ATP6*	[24]
Mitochondrial Encephalo-cardiomyopathy	m.4320C > T	*tRNA^Ile^*	[23]
Mitochondrial Cardiomyopathy	m.10015T > C	*tRNA^Gly^*	-
Mitochondrial Cardiomyopathy	m.12530A > G	*ND5*	-
Mitochondrial Cardiomyopathy	m.1617C > T		-
Mitochondrial Encephalomyopathy	m.6413T > C	*CO1*	-
Mitochondrial Myopathy	m.7608G > A		-
CPEO	Unique deletion	Various gene	[22]
	Multiple deletion		[22]
Kearns Sayre	Unique deletion		[22]
Pearson	Unique deletion		[22]

## 3. Diseases

Nowadays, more than 150 pathogenic mtDNA mutations associated with a range of illnesses have been described in humans. These mutations are identified in more than 30 out of 37 mitochondrial genes. A detailed analysis of the family tree aims to identify minor clinical signs in related parties. There is no risk for the offspring of a man carrying a point mutation. On the other hand, the risk is high for the offspring and siblings of a woman with an mtDNA mutation.

Mitochondrial mutations are heterogeneous and can appear at any age (from antenatal to advanced life) and affect all the tissues. They are mentioned along with an association of symptoms or clinical or paraclinical signs. The difficulty of diagnosis is related to the double genomic control (mitochondrial and nuclear) of the OXPHOS system. For some pathologies the absence of mtDNA mutation in the blood sample does not confirm the diagnosis. It is therefore necessary to perform a tissue biopsy to identify histological lesions of a biochemical deficit of the respiratory chain, as well as a mutation or instability of mtDNA, to allow genetic analysis to be directed [25].

### 3.1. Metabolic Diagnosis

An enzymatic deficit in the respiratory chain causes a profound modification of the cytoplasmic and mitochondrial oxidoreduction balances, by the accumulation of reduced equivalents (*NADH*, *FADH*). In mitochondria, this accumulation of *NADH* leads to the transformation of acetoacetate into 3-hydroxybutyrate resulting in an increase in the ratio of 3-hydroxybutyrate to acetoacetate. Similarly, in the cytoplasm, the transformation of pyruvate into lactate is promoted and the lactate/pyruvate ratio rises with a secondary increase in lactate concentration. More often, the metabolic assessment shows, in children, abnormalities suggestive of mitochondrial mutations, such as persistent hyper-lactacidemia (>2.5 mM), elevated lactate/pyruvate ratio (>20) and ketone body ratio (3-hydroxybutyrate/acetoacetate > 3). This disturbance of the redox balances represents a formal indication of an enzymological exploration in the respiratory chain. Paradoxical hyperketonemia, hyper-lactorachy, hyper-lactaturia, urinary excretion of Krebs ring intermediates or 3-methylglutaconic acid on organic acid chromatography, and elevation of alanine or proline on plasma amino acid chromatography may also occur [26].

### 3.2. Tissue Exploration

Tissue exploration plays an important role in the diagnosis of mitochondrial myopathies. Anatomopathological examination confirms the presence of certain anomalies. Their absence, however, as is often the case in children, does not exclude diagnosis. On muscle biopsy, for example, we look for the presence of ragged-red fibers, but also COX-negative fibers, lipid overload, subsarcolemmal mitochondrial aggregates, and electron microscopy anomalies (globular mitochondria, abnormal mitochondrial ridges) [26].

### 3.3. Enzymatic Diagnosis

The enzymological explorations of the respiratory chain carried out on the affected tissue are studies in polarography of oxygen consumption and spectrophotometry of the enzymatic activity of the complexes. However, the identification of a respiratory chain deficiency allows genetic explorations to be directed towards the gene corresponding to the disease [26].

### 3.4. Genetic Exploration

The term mitochondrial pathology includes conditions related to a dysfunction of the mitochondrial respiratory chain leading to a deficit in mitochondrial oxidation phosphorylation coupling, resulting in a decrease in ATP cell production capacity. These are genetic diseases that affect 2.5 out of 10,000 people, making them the leading metabolic diseases.

The search for the genetic cause of mitochondrial diseases is complex because of the double genetic origin of the genes encoding the protein subunits of the OXPHOS chain complexes and the large number of genes involved: about 80 genes of the protein subunits of the respiratory chain complexes and several hundred nuclear regulations, assembly and maintenance genes necessary for the synthesis, installation and harmonious functioning of the system. Some molecular biology tests are able to look for a depletion either in the muscle or liver, corresponding to a decrease in the quantity of mtDNA, generally less than 10% of the normal value, or multiple deletions of mtDNA. The analyses of these anomalies focus on the nuclear genes involved in mtDNA stability. On the other hand, the presence of a single deletion of mtDNA leads to the molecular diagnosis of Kearns-Sayre or Pearson syndrome [23].

However, when a new mutation is discovered, and to determine if it is the cause of the disease, different functional mitochondrial studies are done using transmitochondrial cybrids cells that are constructed by fusion of platelets of the patient presenting the mutation, with the rho osteosarcoma cell line.

## 4. Mitochondrial Pathologies

More than 120 pathologies of mitochondrial origin have been identified in humans [14]. Pathogenic mutations of mitochondrial DNA can be divided into three categories: rearrangements of mitochondrial DNA, non-synonymous mutations involving genes encoding subunits of respiratory chain complexes, and mutations involving genes related to protein synthesis (ribosomal and transfer RNA). Clinical manifestations associated with mtDNA mutations mainly affect the brain, heart, skeletal muscle, liver and endocrine system. Specific symptoms include some forms of blindness, deafness, motor disorders, cardiovascular disease, muscle weakness, kidney dysfunction and endocrine disorders such as diabetes [16].

Examples of the most frequent mitochondrial pathologies are: Pearson, Kearns-Sayre, CPEO, MELAS, LHON, and MERRF.

### 4.1. Pearson

Pearson syndrome, first described in 1979, is characterized by refractory sideroblastic anemia, vacuolation of marrow precursors and external pancreatic insufficiency. Hematological manifestations usually begin in early childhood and other organ disorders are also possible, including kidney disease with tubulopathy and amino aciduria. Children who do not die in the early years of life usually progress to Kearns-Sayre syndrome. Genetic analysis allows confirmation of the diagnosis by revealing a deletion of mitochondrial DNA in all tissues (at least 1000 base pairs deleted). This syndrome is rarely diagnosed in the neonatal period and must be diagnosed in the presence of sideroblastic macrocytic anemia, which should lead to the rapid performance of a myelogram and then a genetic analysis based on a blood sample [27]. Histological examination shows erythroid precursors with vacuolation typical of hematopoietic precursors, and presence of ring sideroblastic [28].

### 4.2. Kearns-Sayre

Kearns-Sayre syndrome is a multi-systemic disorder that appears early in children and adolescents (before the age of 20). It is defined as pigmentary retinitis, CPEO, cardiac conduction disorders associated with staturo-ponderal delay, cerebellar ataxia, sensorineural hearing loss, diabetes, and severe cognitive impairment. At the molecular level, more than 150 different deletions have been reported in this syndrome and patients with this disease usually die before the age of 40. Muscle biopsy very often shows negative cytochrome oxidase (COX) fibers and “reddish shredded fibers (RRF)” that signify mitochondrial involvement. The search for deletions of mitochondrial DNA confirms the diagnosis [24]. Pigmentary retinopathy is defined by an appearance of fine pigment deposits at the fundus, a variable degree of retinal atrophy and optical atrophy. This is accompanied by a variable degree of night blindness and peripheral visual field impairment [29].

### 4.3. CPEO

CPEO (chronic progressive external ophthalmoplegia) or PEO (progressive external ophthalmoplegia) are characterized by ophthalmoplegia, bilateral ptosis of the eyelids, and myopathy, often accompanied by mtDNA instability. In muscle biopsy, negative COX fibers are present in the muscle, a sign of mitochondrial myopathy. Some patients with a single mtDNA deletion have ocular myopathy of the CPEO type, isolated or associated with peripheral muscle involvement. In general, the disease usually appears in adolescence or in young adults spontaneously and without a family history [30].

In CPEOs and Kearns-Sayre syndrome, deletion is generally found only in muscle while it is present in all tissues in children with Pearson syndrome.

### 4.4. MELAS

MELAS (mitochondrial encephalomyopathy lactic acidosis, and stroke-like episodes), a multi-systemic disorder with onset generally in childhood, is characterized by encephalo-myopathy, lactic acidosis, and recurrent and transient stroke, causing dysfunction of the subacute brain and changes in the brain structure accompanied by hemiparesis, and cortical blindness, as well as many other characteristics such as generalized seizures, migraines, deafness, dementia, vomiting and weakness in the extremities. This syndrome is caused, in more than 80% of cases, by a mutation (m.3243A > G) located in the *tRNALeu* (UUR) gene, but other mutations have also been found in the same *tRNA* [31]. The diagnosis of MELAS includes a check of the lactic acid level in the blood and cerebrospinal fluid and blood tests to check for the presence of an enzyme (creatine kinase) in the muscle of patients. A tissue biopsy is also required for most of the genetic abnormalities present in MELAS. The study of brain images, such as computerized tomography scans (CT) or magnetic resonance imaging (MRI), can detect signs of brain damage [31].

### 4.5. LHON

LHON (Leber’s hereditary optic neuropathy) was the first human disease, along with maternal inheritance, associated with mtDNA damage, specifically the mutation (m.11778G > A) located in the *ND4* gene that causes the most severe form of the disease and is responsible for 50% of cases. However, two other mutations, m.3460G > A and m.14484T > C, located respectively in the genes of *ND1* and *ND6*, are also causes of the appearance of LHON. It is clinically characterized by acute or bilateral subacute optic neuropathy with optic atrophy, sudden loss of central vision, edema of the optical disc, microangiopathy and a major defect of the central visual field. It usually appears in the second or third stage of life and affects men more than women [10].

Without a family history of pathology, the diagnosis of LHON is difficult and generally requires neuro-ophthalmological assessment by angiography and ophthalmoscopy if necessary, as well as blood tests, which are performed by molecular genetic analysis, using PCR (polymerase chain reaction techniques) to detect mutations. The test is 100% accurate for the pathology when visual loss has already occurred. Family members of a patient who tests positive can be symptomatic or asymptomatic and can present a very high-risk factor, so it is important for them to be tested, as a change in lifestyle and adequate diet can help prevent the onset of the disease [32].

### 4.6. MEERF

MERRF syndrome (Myoclonus epilepsy with ragged-red fibers) is an inherited mitochondrial disorder characterized by many clinical signs such as ataxia, myoclonic and generalized epilepsy and myopathy. When a patient with MERRF mutations is suspected based on several clinical manifestations, a diagnosis is initiated, starting with an electroencephalography showing the traces left by generalized seizures, followed by the detection of the presence of reddish fibers tearing at muscle biopsies and deficient COX. However, it has recently been determined that the presence or absence of shredded reddish fibers does not confirm the diagnosis of a MERRF [33]. Molecular genetic analysis is therefore mandatory. This was mentioned in the study by Yeong and his team who confirmed the presence of an A8344G mutation characteristic of a MERRF despite the absence of RRFs, although noting that they may appear later with the evolution of the pathology and the patient’s age [33].

## 5. Therapeutic Approaches

One of the main issues related to mitochondrial diseases is how to treat them [34]. As shown above, these diseases are characterized by extremely heterogeneous symptoms, ranging from organ-specific to multisystemic dysfunction and presenting different clinical courses. This large variability of phenotypical presentations has prevented the development of effective therapies [35]. Until now, all the treatments were directed to alleviating the symptoms that occur due to defects in ATP production. However, in recent years, different pharmaceutical companies have started to develop drugs that, through various mechanisms, could increase the activity of the respiratory chain [36]. Most of these drugs are now under clinical assays and are still not commercially available, although it seems that they have promising preclinical results [37].

Other approaches are specialized in order to treat specific mutations or particular metabolic situations. Among these, there are strategies that include supplementation of nucleotides for thymidine kinase 2 (*TK2*) mutations using deoxi-pyrimidine nucleosides, delivery of nucleic acids to mitochondria, heteroplasmic shift using selective nucleases, allotopic gene expression, etc. [38]. Gene therapy for Leber hereditary optic neuropathy (LHON) is being tried for the m.11778G > A mutation located in the *ND4* gene. Thus, the investigators first carried out preliminary studies to verify the safety and efficacy of gene therapy for LHON by injecting a single vitreous cavity injection of recombinant Adeno-Associated Virus-*NADH* dehydrogenase, subunit 4 (complex I) to the worst-affected eye and noticed that six out of nine patients have vision improvement and no adverse events were observed [39]. Many investigations are in progress into the treatment of mitochondrial diseases and we expect that this field can provide solutions in the next decades.

## 6. Conclusions

Two specificities of mtDNA are important for the understanding of MM: on the one hand, exclusively maternal transmission and, on the other, the notion of heteroplasmy, corresponding to the percentage of mutated mtDNA that varies from one cell type to another. This heterogeneity of the mitochondrial genome makes the diagnosis of MM difficult. This explains the fact that the diagnostic yield of mitochondrial diseases, particularly in children, remains very low because most of the genes responsible are currently unknown and positional cloning strategies are rarely possible (high clinical and genetic heterogeneity, sporadic disease and rarely informative families). Despite these difficulties, the regular discovery of new nuclear genes responsible for mitochondrial cytopathies and access to mtDNA analysis makes it possible to improve this diagnostic management and offer the possibility of genetic counseling and prenatal diagnosis.

Nowadays the contribution of next generation sequencing (NGS) allows sequencing of the entire human genome within a single day and should be considered in the diagnosis of mitochondrial mutations.
